# Can Combining Performance-Based Financing With Equity Measures Result in Greater Equity in Utilization of Maternal Care Services? Evidence From Burkina Faso

**DOI:** 10.34172/ijhpm.2020.121

**Published:** 2020-07-27

**Authors:** Takondwa Mwase, Julia Lohmann, Saidou Hamadou, Stephan Brenner, Serge M.A. Somda, Hervé Hien, Michael Hillebrecht, Manuela De Allegri

**Affiliations:** ^1^Heidelberg Institute of Global Health, Medical Faculty and University Hospital, Heidelberg University, Heidelberg, Germany.; ^2^Department of Global Health and Development, London School of Hygiene & Tropical Medicine, London, UK.; ^3^The World Bank, Yaoundé, Cameroon.; ^4^Centre MURAZ, Bobo-Dioulasso, Burkina Faso.; ^5^UFR/ST, Université Nazi Boni, Bobo-Dioulasso, Burkina Faso.

**Keywords:** Performance-Based Financing, Equity, Equity Measures, Maternal Health Services, Burkina Faso

## Abstract

**Background:** As countries reform health financing systems towards universal health coverage, increasing concerns emerge on the need to ensure inclusion of the most vulnerable segments of society, working to counteract existing inequities in service coverage. To this end, selected countries in sub-Saharan Africa have decided to couple performance-based financing (PBF) with demand-side equity measures. Still, evidence on the equity impacts of these more complex PBF models is largely lacking. We aimed at filling this gap in knowledge by assessing the equity impact of PBF combined with equity measures on utilization of maternal health services in Burkina Faso.

**Methods:** Our study took place in 24 districts in rural Burkina Faso. We implemented an experimental design (clusterrandomized trial) nested within a quasi-experimental one (pre- and post-test design with independent controls). Our analysis relied on self-reported data on pregnancy history from 9999 (baseline) and 11 010 (endline) women of reproductive age (15-49 years) on use of maternal healthcare and reproductive health services, and estimated effects using a difference-in-differences (DID) approach, purposely focused on identifying program effects among the poorest wealth quintile.

**Results:** PBF improved the utilization of few selected maternal health services compared to status quo service provision. These benefits, however, were not accrued by the poorest 20%, but rather by the other quintiles. PBF combined with equity measures did not produce better or more equitable results than standard PBF, with specific differences only on selected outcomes.

**Conclusion:** Our findings challenge the notion that implementing equity measures alongside PBF is sufficient to produce an equitable distribution in program benefits and point at the need to identify more innovative and contextsensitive measures to ensure adequate access to care for the poorest. Our findings also highlight the importance of considering changing policy environments and the need to assess interferences across policies.

## Background

**Key Messages**

** Implications for policy makers**

- Our study detects no clear added benefit of the equity measures implemented in conjunction with PBF compared to stand-alone performance-based financing (PBF).
- The experience of the PBF program in Burkina Faso provides a clear illustration of how even well-intended and accurately designed interventions may fail to achieve their objective due to a variety of contextual elements that shape implementation in unattended manners.
- Greater attention to context and competing policies ought to be paid in designing strategies that aim at building synergies between supply and demand to overcome existing inequities.

** Implications for the public**

Performance-based financing (PBF) is being implemented across sub-Saharan Africa, as a presumed means of strengthening health systems performance, but concerns persist that PBF may reinforce instead of counteracting existing inequalities in access to and utilization of health services. As such, selected countries are coupling PBF with targeted demand-side interventions aimed at increasing access for the most vulnerable segments of the population. Yet evidence on the effects of these combined interventions is extremely limited. The study investigated the equity impact of PBF combined with equity measures on utilization of maternal health services. Our findings show that PBF combined with equity measures does not lead to improved equity in utilization of maternal health services and points at the need to carefully consider the context and complementary policies so as to build synergies between demand and supply in order to reduce inequities in maternal health services utilization.

There is a growing concern that health inequalities related to social determinants of health are responsible for the slow progress witnessed in health and healthcare at global, regional and country levels, potentially jeopardizing opportunities to achieve the health related Sustainable Development Goals (SDGs).^1^ As countries embark on health financing and service delivery reforms, often targeting women and children first, monitoring health and healthcare inequalities remains an essential element of tracking progress towards the SDG 3 target of achieving universal health coverage by 2030, ensuring that no one is left behind.^2^ Monitoring is even more urgent and important in low- and middle-income countries (LMICs) because, despite the recent progress made in curbing maternal and child deaths, serious inequities in maternal and child health persist, especially in sub-Saharan Africa.^3^ Exacerbating the situation, LMICs also face huge coverage gaps, health system inefficiencies, and insufficient quality of service delivery.^4^

In recent years, much attention has been paid to performance-based financing (PBF) as a possible strategy to improve health system performance. PBF aims at reorienting health providers’ behavior towards provision of more and better quality care through the implementation of performance contracts that reward the attainment of predefined targets.^5^ One vividly debated program design topic is a PBF programs’ potential to reinforce rather than to counteract existing inequities.^6,7^ Still, very few studies have looked at the equity impact of PBF on health services use and maternal care services in particular. While some studies suggest pro-least poor effects,^8,9^ others find evidence for the opposite^10^ or distributional-neutral effects.^11,12^ Given the mixed evidence, an increasing number of authors advocate the introduction of PBF designs deliberately aimed at spreading program benefits more evenly across wealth groups.^8,11,12^ However, to date limited evidence is available.^13^

Our study evaluates an “equity-conscious” PBF design, which was recently implemented in Burkina Faso and which was piloted with 3 different equity measures. We examined program effects on maternal health service utilization and defined equity as equal access given equal need, with access measured as reported service use and need,^14^ measured in terms of a woman’s pregnancy status.

## Methods

###  Study Setting 

Burkina Faso is a landlocked West African country with a population of 18.6 million and a life expectancy of 60 years. Infant and under-five mortality rates stand respectively at 61 and 89 deaths per 1000 live births. An estimated 41.1 percent of the population live below the national poverty line of US$1.90 a day. Maternal mortality remains high at an estimated 371 per 100 000 live births. Multiple challenges related to maternal care persist, including serious inequities in access linked to sub-standard quality, low geographical accessibility, and financial barriers.^15^

Prior to PBF, Burkina Faso undertook several health financing reforms to increase coverage and reduce inequities in access to and utilization of maternal health services, such as removal of user fees for antenatal care (ANC) services in 2002, and an 80% removal of user fees for delivery care in 2007, with a provision for full exemption of the ultra-poor.^16^ Later in 2016, with the introduction of national free healthcare policy, known as the *gratuité*, the government removed all user fees for services delivered to children under the age of 5 years and to pregnant and lactating women.^17^ As described below, the introduction of the national free healthcare policy induced the Ministry of Health to modify PBF prices, specifically to remove the equity measure additional payments (implemented in PBF2 and PBF3) for selected services targeted by both the national free healthcare policy and the PBF program.^18^

###  The Intervention and the Study Design 

Following an initial pilot in the districts of Titao, Leo, and Boulsa, starting in January 2014, Burkina Faso piloted PBF combined with different equity interventions in 12 districts distributed across 6 regions (Boucle du Mouhoun, Centre-Nord, Centre-Ouest, Nord, Sud-Ouest, Centre-Est) in which health facilities were rewarded by the Ministry of Health for achievement of defined health service indicators using a case-based payment system, adjusted for quality of care after verification. More details on the intervention design have been described elsewhere.^18^ In brief, PBF was implemented according to 4 different models, 3 of which included an equity intervention targeting specifically the ultra-poor, as summarized in Table 1. The details of the ultra-poor selection process have been described elsewhere.^19,20^

**Table 1:** Performance-Based Financing Models and Their Description

**PBF Model**	**Description**
Standard PBF (PBF1)	Performance contracts based on case-based payments method adjusted for quality were signed between the Ministry of Health and health facilities. Verification agencies were employed to verify service provision data submitted by individual facilities. PBF unit prices were calculated based on the relative cost and frequency of the services provided. Additional incentives were calculated on the basis of quantity outcomes and service quality if facilities achieved a quality score of 50% (and later changed to 60%), every quarter. Incentives were expected to pay for expenditures incurred, to increase savings and to pay bonuses to individual staff members.
PBF1 plus systematic targeting and subsidization of health services for the ultra-poor (PBF2)	Used the same health service purchasing model as PBF1, but had specific equity measures meant to ease access to and utilization of maternal healthcare services among the ultra-poor living in the catchment areas of the participating health facilities with the following components: (*a*) a systematic targeting of the ultra-poor to identify a maximum of poorest 20% of the population; (*b*) providing the identified ultra-poor with proof of status so they could access health services at no cost at the point of use; and (*c*) higher purchase unit prices than in PBF1 for health services delivered to the targeted ultra-poor (ie, as compensation for the lost revenues due to free health services provided to the ultra-poor at the point of use). The adjusted higher unit prices were only for services where user fees existed, such as tetanus toxoid vaccine, delivery and family planning services among others, while for services already provided free of charge at point of use, such as HIV and tuberculosis testing and treatment among others, the same unit prices as in standard PBF were used. The additional payments were removed in June 2016 after the introduction of the national free healthcare policy.
PBF2 combined with higher incentive purchase price to provide health services to the ultra-poor (PBF3)	Used the same purchasing arrangement as PBF1 and PBF2 and also involved the same targeting mechanisms and equity measures for the ultra-poor as in PBF2. The main difference was in the unit prices, whereby services provided to the ultra-poor were reimbursed at a higher unit price than in PBF2—at around 150% of the PBF2 unit prices. The higher unit prices were meant to compensate for the lost revenue from user fees, and also to offer health workers an additional incentive to motivate them to attract or reach out to the ultra-poor. This applied only to services where user fees were still charged at the point of use. These additional payment were removed in June 2016 after the introduction of the national free healthcare policy.
PBF1 plus community-based health insurance, combined with targeting and subsidization of health services for the ultra-poor (PBF4)	Involved implementation of PBF1 alongside CBHI whereby an annual insurance premium of 3900 F CFA (US$7) per individual was offered for the whole population using the same targeting mechanism as in PBF2 and PBF3. CBHI insurance premiums for the ultra-poor was fully paid for by the PBF program and payments to health facilities were made by both the CBHI scheme as a replacement for user fees and by the PBF program, using a case-based payment system as in PBF1.

Abbreviations: PBF, performance-based financing; CBHI, Community-based health insurance.

 Policy-makers expected the equity measures to induce increased utilization among the ultra-poor through 4 different pathways. First, they assumed that the targeting process would sensitize communities and particularly the targeted ultra-poor to the importance of health service utilization in case of need. Second, they assumed that the equity component would sensitize health workers to the importance of making specific efforts to facilitate health service utilization among the ultra-poor. Third, the removal of user fees for the targeted ultra-poor was assumed to reduce barriers to healthcare utilization among the ultra-poor. And finally, the elevated price levels for treating the targeted ultra-poor patients were assumed to enable and motivate health facilities to provide services to the ultra-poor free of charge.

 To address our study primary objective of measuring the equity impact of PBF combined with equity measures compared to standard PBF alone, we inevitably needed to investigate the effect of PBF compared to status-quo service provision in the first place. To do so, we adopted a design that combined experimental with quasi-experimental elements. More specifically, we conducted a cluster-randomized trial nested within a pre-and post-test study with independent controls. Hereafter, we describe the different elements of our study in detail, referring to the quasi-experiment as study component 1 and to the cluster-randomized trial as study component 2. Figure provides a summary of the details of both study components including final sample sizes used in the analysis.

**Figure:**
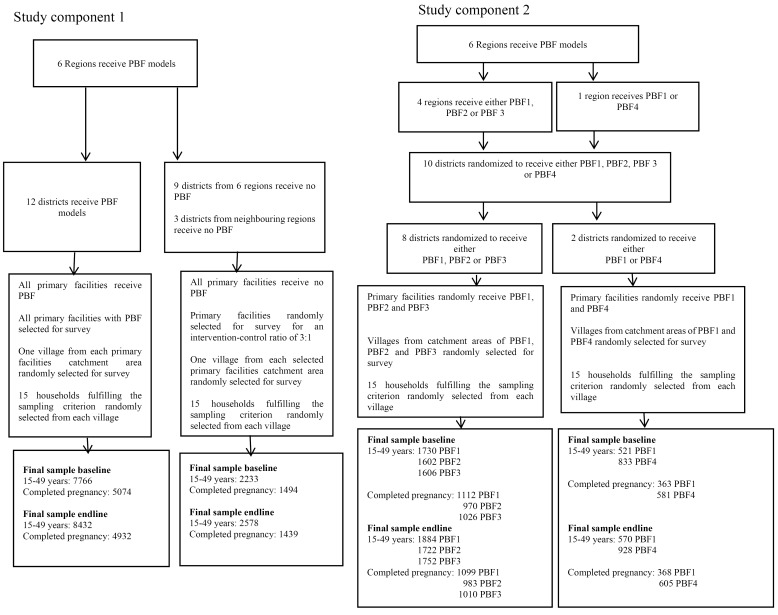
Summary of Study Designs and Sampling Procedures. Abbreviation: PBF, performance-based financing.

####  Study Component 1

For the study component 1, six regions (Boucle du Mouhoun, Centre-Nord, Centre-Ouest, Nord, Sud-Ouest, Centre-Est) were identified non-randomly by the government and its development partners as intervention regions. Within each region, 2 districts were selected as intervention districts, ie, destined to receive PBF, and 2 districts (when not possible in a neighbouring region) as control, ie, continue with *status quo* service provision with no PBF. The intervention districts were purposely selected based on poor performance on selected maternal health indicators.^18^ Control districts were selected to be as similar as possible (also in terms of performance on maternal health indicators) to intervention districts. This study component was set to allow a comparison between PBF districts (12) and districts (12) with status quo service provision without PBF. Since the intervention was assigned at district levels, districts effectively functioned as clusters for this study component.

####  Study Component 2

 For the study component 2, ten out of 12 districts (2 with community-based health insurance (CBHI) where CBHI was pre-existing to allow implementation) were targeted by the Ministry of Health and development partners for randomization due to financial constraints (not enough funds to allow targeting across all 12 selected districts once calculations for targeting costs were made). In 8 out of 10 targeted districts in 4 regions (Centre-East, Centre-Nord, Sud-Ouest, Nord), clusters (primary healthcare facilities) were randomized to receive either PBF1 or PBF combined with either one of 2 equity measures, PBF2, and PBF3 so as to test the additional effect of combining standard PBF with equity measures as one way of reducing inequities in access to and utilization of health services.

Randomization took place within the framework of public randomization ceremonies in which concerned district and regions took turns in drawing primary healthcare facility names from a box containing all primary healthcare facility names in the 4 regions (Centre-East, Centre-Nord, Sud-Ouest, Nord) starting with the selection of pre-defined PBF model, and followed by the assignment of primary healthcare facilities in the order in which they were drawn from the box.^18^ For example, in the 8 districts of the 4 regions (Centre-East, Centre-Nord, Sud-Ouest, Nord), this was done as follows: first facility: PBF1, second facility: PBF2; third facility: PBF3, fourth facility: PBF1, fifth facility: PBF2; and sixth facility: PBF3; etc. In these 8 districts of the 4 regions (Centre-East, Centre-Nord, Sud-Ouest, Nord) concerned by the three-arm randomization, this resulted into samples of 90 PBF1 facilities, 83 PBF2 facilities, and 84 PBF3 facilities. In the Boucle du Mouhoun region where 2 districts already implementing CBHI were targeted, 59 facilities were randomized to receive either PBF1 or PBF4 (following the same procedure outlined above), generating samples of 29 PBF1 and 30 PBF4 facilities. The 18 facilities which had been implementing CBHI prior to the launch of the study were excluded from randomization and hence from our study. Nevertheless, for ethical reasons, these facilities all implemented PBF in addition to CBHI. This study component was set to allow us to measure the benefit of combining PBF with an equity measure compared to implementing standard PBF on its own. In particular, we used this experimental component to measure the additional equity effects of PBF2, PBF3, and PBF4 compared to PBF1.

###  Sampling and Data Sources 

 For both study components, we used repeated cross-sectional household survey data collected at baseline from November 2013-March 2014 and at endline from April-June 2017. Sampling followed a three-stage cluster sampling procedure. First, for each primary healthcare facility included in the study (416 in intervention districts and 117 in the control districts — the number of facilities included in the study is larger for intervention compared to control districts since in intervention districts, we took a census of all facilities while in control districts we randomly selected one third of all facilities), we randomly selected one village. Second, within each village, we randomly selected 15 out of all households identified in each village where at least one woman was pregnant or had completed a pregnancy in the prior 24 months (inclusion criteria). Third, within a household, we interviewed all women of reproductive age (15-49 years), irrespective of whether they had a recent history of pregnancy.

 The survey collected information on use of reproductive and maternal health services from women of reproductive age (15-49 years). Data on use of family planning were collected from all women of reproductive age regardless of marital status while data on use of maternal health services were collected only from women with a recent pregnancy. At baseline in study component 1, our sample comprised 9999 (7766 in intervention group, 2233 in control group) women of reproductive age (15-49 years) of whom 6568 (5074 in intervention group, 1494 in control group) had completed pregnancy 24 months prior to the survey. For the study component 2, at baseline, our sample comprised 6292 (1730 in PBF1, 1602 in PBF2, 1606 in PBF3 in the 4 regions [Centre-East, Centre-Nord, Sud-Ouest, Nord] and 521in PBF1 and 833 in PBF4 in Boucle du Mouhoun) women of reproductive age (15-49 years) of whom 4052 (1112 in PBF1, 970 in PBF2, 1026 in PBF3 in the 4 regions [Centre-East, Centre-Nord, Sud-Ouest, Nord] and 363 in PBF1 and 581 in PBF4 in Boucle du Mouhoun) had completed pregnancy 24 months prior to the survey. At endline, in study component 1, our sample comprised 11 010 (8432 in intervention group, 2578 in control group) women of reproductive age (15-49 years) of whom 6371 (4932 in intervention group, 1439 in control group) had completed pregnancy 24 months prior to the survey. For the study component 2, at endline, our sample comprised 6856 (1884 in PBF1, 1722 in PBF2, 1752 in PBF3 in the 4 regions [Centre-East, Centre-Nord, Sud-Ouest, Nord], and 570 in PBF1 and 928 in PBF4 in Boucle du Mouhoun) women of reproductive age (15-49 years) of whom 4065 (1099 in PBF1, 983 in PBF2, 1010 in PBF3 in the 4 regions [Centre-East, Centre-Nord, Sud-Ouest, Nord], and 368 in PBF1 and 605 in PBF4 in Boucle du Mouhoun) had completed pregnancy 24 months prior to the survey.

###  Variables and Their Measurement

Table 2 summarizes all outcomes and control variables. Our outcomes were selected to capture service coverage (defined as utilization given need, ie, pregnancy status) along the reproductive and maternal health service continuum and to reflect services which were incentivized by the PBF program, namely: ANC in the first trimester, at least 4 antenatal care (ANC4+) visits, at least 2 doses of tetanus toxoid vaccine (TTV2+), iron supplementation, HIV testing in pregnancy, facility-based delivery, at least 1 postnatal care (PNC1+) visit, at least 3 postnatal care (PNC3+) visits, and modern family planning methods (female sterilization, male sterilization, intrauterine device [IUD]/spiral, injectables/depoprovera, implants/norplant, male condom, female condom, diaphragm, foam/jelly). To improve the estimation precision, we included a number of control variables, which have the potential to explain the variation in outcome indicators from our previous work.^21^ We relied on multiple correspondence analysis — run separately on baseline and endline samples —to generate a wealth index based on asset ownership and dwelling characteristics.^22^ Given our specific research focus and the intervention’s intention to treat the ultra-poor^23^ and in line with prior literature,^8^ we divided households in 2 wealth brackets corresponding to the Lowest 20% (ie, ultra-poor) and the rest — Upper 80%.

**Table 2:** Variables and Their Measurement

	**Measurement**
**Outcome variables**	
ANC visit in the first trimester	1 = Attended first antenatal care consultation within the first trimester
	0 = Attended first antenatal care consultation later than first trimester
At least 4 ANC visits (ANC4+)	1 = Attended at least 4 antenatal care consultations
	0 = Attended less than 4 antenatal care consultations
At least 2 doses of tetanus toxoid vaccine (TTV2+)	1 = Received at least 2 tetanus doses during pregnancy
	0 = Received less than 2 doses during pregnancy
HIV testing in pregnancy	1 = Received HIV testing during pregnancy
	0 = Did not receive HIV testing during pregnancy
Iron supplementation	1 = Received iron supplements during pregnancy
	0 = Did not receive iron supplements during pregnancy
Facility-based delivery	1 = Delivered at a formal health facility
	0 = Did not deliver at a formal health facility
At least 1 postnatal care visit (PNC1+)	1 = Attended at least one postnatal care consultation within 6 weeks post-delivery
	0 = Did not attend any postnatal care consultations within 6 weeks post-delivery
At least 3 postnatal care visits (PNC3+)	1 = Attended at least 3 postnatal care consultations within 6 weeks post-delivery
	0 = Attended less than 3 postnatal care consultations within 6 weeks post-delivery
Modern family planning^a^	1 = Used any modern family planning method
	0 = Used no or traditional family planning method
**Control variables**	
Household wealth	0 = Lowest 20%
	1 = Upper 80%
Marital status	0 = Unmarried
	1 = Married
Literacy	0 = Illiterate
	1 = Literate
Age	0 = 15-20 years
	1 = >21 years
Parity	0 = 0-3 pregnancies
	1 = ≥4 pregnancies
Distance to catchment health centre	0 = >5 km to catchment health centre
	1 = ≤5 km to catchment health centre

Abbreviations: ANC, antenatal care; TTV, tetanus toxoid vaccine; PNC, postnatal care. ^a^Includes female sterilization, male sterilization, intrauterine device (IUD)/spiral, injectables/depoprovera, implants/norplant, male condom, female condom, diaphragm, foam/jelly.

###  Data Analysis 

####  Bivariate Analysis

First, we used *t* tests to assess systematic differences in the distribution of Outcome variables and Control variables across study arms for both study components.

####  Regression Analysis

Second, to assess the overall impact of PBF compared to status quo, we relied on study component 1 and used a difference-in-differences (DID) estimation approach,^24^ comparing intervention districts (irrespective of specific study arm) with control districts. We estimated a linear probability model, where we clustered standard errors at district level. In addition, for each outcome, we included village fixed effects and several individual-level covariates (equation 1):


(1) $$Ydvit=\alpha v+\beta .Y17t+\beta .PBFd+\lambda .\left[PBFd*Y17t\right]+\delta .Xit+\varepsilon dvit$$

where *Y*_dvit_ outcome for individual *i* from villagev in district *d* at time *t* with *t *as* (baseline, endline ); Y17*_t_ is dummy variable representing endline; *PBF*_d_ is dummy variable denoting PBF exposure (1 = PBF, 0 = control); *α*_v_ is village fixed effects capturing time-invariant unobserved differences across villages. *X*_it_ is vector of individual-level covariates; and ε_dvit_ is error term. *λ* is the variable of interest (interaction term between PBF and endline) that gives the DID estimate for the effect of being located in a PBF district.

To determine overall PBF effects compared to status quo by socio-economic status group, we estimated regression model 1 by wealth bracket, following Lannes et al.^8^

Third, to answer our key question on the equity impact of the PBF models integrating equity interventions, we relied exclusively on study component 2 of our study (10 districts) and also used DID to estimate a linear probability model as in equation 1, but with standard errors clustered at village level where randomization occurred. In this study component, equation 2 and equation 3 pertain, respectively, to the 8 districts in 4 regions (Centre-East, Centre-Nord, Sud-Ouest, Nord) where PBF1, PBF2, and PBF3 were randomized and to the 2 districts in Boucle du Mouhoun region where PBF1 and PBF4 were randomized:


(2) $$Yvit=\alpha v+\beta .Y17t+\beta .PBF2v+\lambda 2.\left[PBF2v*Y17t\right]+\beta .PBF3v+\lambda 3.\left[PBF3v*Y17t\right]+\delta .Xit+\varepsilon vit$$

(3) $$Yvit=\alpha v+\beta .Y17t+\beta .PBF4v+\lambda 4.\left[PBF4v*Y17t\right]+\delta .Xit+\varepsilon vit$$

where *Y*_vit_ is outcome for individual *i* from village* v* at time *t* with *t *as *(baseline, endline )* in the intervention districts. *λ*_2_ and *λ*_3_ are variables of interest that give the DID estimates for the effects of being resident in PBF2 and PBF3 compared to PBF1, respectively, and *λ*_4_ is the variable of interest that give the DID estimate for the effect of being resident in PBF4 compared to PBF1 in the Boucle du Mouhoun region.

Similarly to what we described earlier, to estimate specific effects by socio-economic status, we performed separate analyses by wealth bracket.^8^

Furthermore, for study component 1, we performed several robustness checks to account for the small number of clusters (24 districts). We did so in light of the existing literature suggesting that: (1) a small number of clusters results in a higher likelihood of estimating downwards-biased standard errors, potentially leading to over rejection of the null hypothesis, ie, suggesting significant program impact while in reality there is none or very little impact^25^; and (2) bias arising from a small number of clusters is more acute in situations characterized by an imbalance in cluster sample sizes.^26^ Hence, to account for these 2 problems pertaining to our study component 1, we relied on the ‘wild bootstrap’ method for the related analyses. This method relies on a bootstrap t- procedure instead of bootstrapping the standard errors.^25^

 We performed all analyses using Stata14 (Stata Corporation, Texas, USA).

## Results

 The results of our study are presented according to study components as follows:

###  Study Component 1

####  Bivariate Analysis

Tables 3 and 4 shows bivariate analysis of the characteristics of women of reproductive age (15-49 years) and coverage of maternal health services at baseline, respectively. At baseline, women in intervention and control districts were comparable on most demographic characteristics except age (Table 3).

**Table 3:** Comparison of Women Characteristics Between PBF and Control Samples at Baseline

**PBF vs. Control**					
**Control Variable**	**Total**		**PBF**	**Control**	**Difference**
	**N = 9999**	**Mean (%)**	**Mean (%)**	**Mean (%)**	
Age					
15-20 years	1918	19.18	20.10	15.99	4.11***
>21 years	8081	80.82	79.90	84.01	
Literacy					
Literacy	1320	13.20	13.21	13.17	0.05
Illiteracy	8679	86.80	86.79	86.83	
Parity					
0-3 pregnancies	5911	59.12	59.46	57.90	1.56
≥4 pregnancies	4088	40.88	40.54	42.10	
Marital status					
Married	9431	94.32	94.48	93.78	0.70
Unmarried	568	5.68	5.52	6.22	
Distance					
1 ≤5 km	6097	60.98	61.47	59.25	2.23
0 = >5 km	3902	39.02	38.53	40.75	
Wealth groups					
Lowest 20%	1765	17.65	17.37	18.63	-1.26
Upper 80%	8234	82.35	82.63	81.37	

Abbreviation: PBF, performance-based financing. *T* test for differences between PBF and control samples; *** *P* < .01.

**Table 4:** Comparison of Utilization of Maternal Health Services Between PBF and Control Samples at Baseline and Endline

**Outcome Variable**	**Baseline**							**Endline**						
	**Total**		**PBF**		**Control**		**Difference**	**Total**		**PBF**		**Control**		**Difference**
	**n**	**Mean (%)**	**n**	**Mean (%)**	**n**	**Mean (%)**		**n**	**Mean (%)**	**n**	**Mean (%)**	**n**	**Mean (%)**	
ANC 1^st^ trimester	6357	67.17	4909	67.39	1448	66.44	0.95	6168	73.98	4773	75.63	1395	68.32	7.31***
ANC4+^a^ visits	6564	44.20	5070	44.91	1494	41.77	3.14**	6365	61.62	4928	62.50	1437	58.59	3.91***
TTV2+^b^	6557	62.80	5065	62.65	1492	63.34	-0.69***	6213	43.68	4810	43.10	1403	45.69	-2.59*
HIV testing in pregnancy	6557	56.11	5065	57.14	1492	52.61	4.53	6215	54.88	4812	56.44	1403	49.54	6.90***
Iron supplementation	6557	93.18	5065	92.99	1492	93.83	-0.84	6209	98.20	4807	98.25	1402	98.00	0.25
Facility-based delivery	6511	89.08	5024	88.42	1487	91.32	-2.90***	6220	91.27	4807	91.72	1413	89.74	1.98**
PNC1+^c^ visit	6488	53.21	5002	54.48	1486	48.92	5.56***	6194	76.40	4785	77.93	1409	71.19	6.74***
PNC3+^d^ visits	6488	5.50	5002	4.96	1486	7.34	-2.38***	6194	13.63	4785	14.48	1409	10.72	3.76***
Modern planning family methods	8643	11.25	6814	11.43	1829	10.55	0.88	8942	27.25	6806	27.80	2136	25.51	2.29

Abbreviations: TTV, tetanus toxoid vaccine; PNC, postnatal care; PBF, performance-based financing; ANC, Antenatal care. ^a^At least 4 antenatal care; ^b^At least 2 tetanus doses during pregnancy; ^c^At least one postnatal care visit within 6 weeks; ^d^At least 3 postnatal care visits within 6 weeks *T* test for differences between PBF and control samples; * *P* < .1, ** *P* < .05, *** *P* < .01.

In contrast, significant differences between PBF and control group existed in baseline values for a number of outcome variables: ANC4+ visits, HIV testing in pregnancy, facility-based delivery, PNC1+ visit and PNC3+ visits (Table 4).

####  Regression Analysis

Table 5 summarizes the results of the regression models pertaining to the overall impact of PBF compared to status quo, both for the entire sample and stratified by socio-economic group for the study component 1. We detected a positive effect of PBF on utilization of facility-based delivery [4.4 percentage points (pp) (*P* < .1)] and for PNC3+ visits: [6.6 pp (*P* < .1)]. This effect was primarily driven by an effect among the upper 80% of 5.5 pp (*P* < .05) and of 7.2 pp (*P* < .1) for facility-based delivery and PNC3+ visits, respectively. Among the poorest 20%, we detected an increase attributable to PBF for utilization of modern family planning methods of 7.6 pp (*P* < .1).

**Table 5:** DID Estimates of Overall PBF Impacts on the Utilization of Maternal Health Services Compared to Status Quo in the Entire Population as Well as by Wealth Subgroups

**Outcome Variable**	**Full Sample**		**Wealth Subgroups**			
			**Lowest 20%**		**Upper 80%**	
	**Beta**	**Robust Standard Error**	**Beta**	**Robust Standard Error**	**Beta**	**Robust Standard Error**
ANC 1st trimester	0.057	0.056	0.031	0.053	0.059	0.056
ANC4+^a^ visits	-0.004	0.041	0.037	0.067	-0.022	0.037
TTV2+^b^	-0.027	0.063	0.026	0.054	-0.025	0.068
HIV testing in pregnancy	0.001	0.063	0.047	0.092	-0.011	0.060
Iron supplementation	0.012	0.019	0.015	0.034	0.009	0.017
Facility-based delivery	0.044*	0.022	0.022	0.038	0.055**	0.020
PNC1+^c^ visit	0.030	0.064	-0.006	0.096	0.047	0.062
PNC3+^d^ visits	0.066*	0.036	0.041	0.042	0.072*	0.038
Modern family planning methods	0.019	0.024	0.076*	0.040	0.011	0.025

Abbreviations: TTV, tetanus toxoid vaccine; PNC, postnatal care; DID, difference-in-differences; PBF, performance-based financing; ANC, antenatal care. ^a^At least 4 antenatal care; ^b^At least 2 tetanus doses during pregnancy; ^c^At least one postnatal care visit within 6 weeks; ^d^At least 3 postnatal care visits within 6 weeks.* *P* < .1, ** *P* < .05.

Table 6 summarizes the results of the robustness tests —using the “wild bootstrap” method.^25^ The results show that the estimates included in this study were all within the 95% confidence interval, and as such, there is no concern that the DID estimates in this study component are substantially biased due to the small number of clusters and/or to imbalances in cluster sample sizes. These results imply that we are not at risk of detecting an effect as significant when there was in fact no effect.

**Table 6:** Results of “Wild Bootstrap” Method

**Outcome Variable**	**Full Sample**	**Wealth Subgroups**	
		**Lowest 20%**	**Upper 80%**
ANC first trimester			
DID estimate	0.057	0.031	0.059
Wild bootstrap CI-left	-0.031	-0.012	-0.036
Wild bootstrap CI-right	0.173	0.130	0.183
ANC4+^a^ visits			
DID estimate	-0.004	-0.037	-0.022
Wild bootstrap CI-left	-0.047	-0.030	-0.052
Wild bootstrap CI-right	0.124	0.166	0.134
TTV2+^b^			
DID estimate	-0.027	0.026	-0.025
Wild bootstrap CI-left	-0.152	-0.104	-0.179
Wild bootstrap CI-right	0.096	0.146	0.095
HIV testing in pregnancy			
DID estimate	0.001	0.047	-0.011
Wild bootstrap CI-left	-0.098	-0.052	-0.011
Wild bootstrap CI-right	0.023	0.284	0.237
Iron supplementation			
DID estimate	0.012	0.015	0.009
Wild bootstrap CI-left	-0.006	-0.018	-0.010
Wild bootstrap CI-right	0.012	0.043	0.011
Facility-based delivery			
DID estimate	0.044	0.022	0.055
Wild bootstrap CI-left	-0.027	-0.054	-0.027
Wild bootstrap CI-right	0.076	0.122	0.070
PNC1+^c^ visit			
DID estimate	0.030	-0.006	0.047
Wild bootstrap CI-left	-0.030	-0.093	-0.018
Wild bootstrap CI-right	0.077	0.219	0.169
PNC3+^d^ visits			
DID estimate	0.066	0.041	0.072
Wild bootstrap CI-left	-0.024	-0.067	-0.017
Wild bootstrap CI-right	0.100	0.102	0.109
Modern family planning methods			
DID estimate	0.019	0.076	0.011
Wild bootstrap CI-left	-0.030	-0.046	-0.031
Wild bootstrap CI-right	0.077	0.156	0.070

Abbreviations: TTV, tetanus toxoid vaccine; PNC, postnatal care; DID, difference-in-differences; ANC, antenatal care. ^a^At least 4 antenatal care; ^b^At least 2 tetanus doses during pregnancy; ^c^At least one postnatal care visit within 6 weeks; ^d^At least 3 postnatal care visits within 6 weeks.

###  Study Component 2

####  Bivariate Analysis

Tables 7 and 8 presents bivariate analysis of the characteristics of women of reproductive age (15-49 years) and coverage of maternal health services, respectively at baseline in PBF2, PBF3 and PBF1 in the 4 regions (Centre-East, Centre-Nord, Sud-Ouest, Nord) and PBF4 and PBF1 in Boucle du Mouhoun region. At baseline, women across the 4 PBF arms were comparable except for marital status, distance to primary healthcare facilities, and age (Table 7).

**Table 7:** Comparison of Characteristics of Women of Reproductive Age (15-49 Years) Between PBF2, PBF3 and PBF4 Compared With PBF1 at Baseline

**Control Variable**	**PBF2 vs PBF1**					**PBF3 vs. PBF1**					**PBF4 vs. PBF1**				
	**Total**		**PBF2**	**PBF1**	**Difference**	**Total**		**PBF3**	**PBF1**	**Difference**	**Total**		**PBF4**	**PBF1**	**Difference**
	**n**	**Mean (%)**	**Mean (%)**	**Mean (%)**		**n**	**Mean (%)**	**Mean (%)**	**Mean (%)**		**N**	**Mean (%)**	**Mean (%)**	**Mean (%)**	
Age															
15-20 years	687	20.62	21.72	19.60	2.12	666	19.96	20.36	19.60	0.76	279	20.61	22.45	17.66	4.79**
>21 years	2645	79.38	78.28	80.40		2670	80.04	79.64	80.40		1075	79.39	77.55	82.34	
Literacy															
Literate	468	14.05	12.86	15.14	-2.28	480	14.39	13.57	15.14	-1.57	102	7.53	7.68	7.29	0.39
Illiterate	2864	85.95	87.14	84.86		2856	85.61	86.43	84.86		1252	92.47	92.32	92.71	
Parity															
0-3 Pregnancies	1972	59.18	60.61	57.86	2.75	1942	58.21	58.59	57.86	0.73	892	65.88	67.23	63.72	3.51
≥4 Pregnancies	1360	40.82	39.39	42.14		1394	41.79	41.41	42.14		462	34.12	32.77	36.28	
Marital status															
Married	3172	95.20	96.07	94.39	1.68***	3173	95.11	95.89	94.39	1.50***	1326	97.93	97.24	99.04	-1.80***
Unmarried	160	4.80	3.93	5.61		163	4.89	4.11	5.61		28	2.07	2.76	0.96	
Distance to catchment facility															
≤5 km	2307	69.24	66.79	71.50	-4.71***	2279	68.32	64.88	71.50	-6.62***	2307	41.51	36.85	48.94	-12.09***
>5 km	1025	30.76	33.21	28.50		1057	31.68	35.12	28.50		1025	58.49	63.15	51.06	
Wealth groups															
Lowest 20%	514	15.53	14.79	16.01	-1.22	563	16.88	17.81	16.01	1.80	328	24.22	24.97	23.03	1.94
Upper 80%	2818	84.57	85.21	83.99		2773	83.12	82.19	83.99		1026	75.78	75.03	76.97	

Abbreviation: PBF, performance-based financing. *T* test for differences between PBF2 and PBF1, PBF3 and PBF1 “in 4 regions” and PBF4 and PBF1 “in Boucle du Mouhoun” samples. ** *P* < .05, *** *P* < .01.

**Table 8:** Comparison of Utilization of Maternal Health Services Between PBF2, PBF3 and PBF4 Compared With PBF1 at Baseline and Endline

**Outcome variable**	**Time**	**PBF2 vs PBF1**			**PBF3 vs BF1**			**PBF4 vs PBF1**		
		**PBF2** **Mean (%)**	**PBF1** **Mean (%)**	**Difference**	**PBF2** **Mean (%)**	**PBF1 ** **Mean (%)**	**Difference**	**PBF2** **Mean (%)**	**PBF1 ** **Mean (%)**	**Difference**
ANC 1stTrimester	Baseline	71.46	66.91	4.55**	69.01	66.91	2.10	66.97	63.04	3.93
	Endline	80.11	79.98	0.13	81.01	79.98	1.03	69.54	68.52	1.02
ANC4+^a^ visits	Baseline	43.61	43.47	0.14	42.24	43.47	-1.23	48.97	48.48	0.49
	Endline	65.68	68.37	-2.69	64.06	68.37	-4.31**	53.72	54.50	-0.78
TTV2+^b^	Baseline	63.05	59.41	3.64*	54.54	59.41	-4.87**	67.88	68.23	-0.35
	Endline	39.13	42.51	-3.38	42.80	42.51	0.29	42.02	32.96	9.06***
HIV testing in pregnancy	Baseline	58.93	60.04	-1.11	59.61	60.04	-0.43	74.96	69.89	5.07*
	Endline	65.04	62.94	2.10	61.12	62.94	-1.82	47.68	36.21	11.47***
Iron supplementation	Baseline	95.36	94.69	0.67	95.32	94.69	0.63	86.87	89.50	-2.63
	Endline	97.50	98.15	-0.65	98.57	98.15	0.42	98.80	99.72	-0.92
Facility-based delivery	Baseline	90.40	86.47	3.93***	89.88	86.47	3.41**	86.28	88.37	-2.09
	Endline	93.54	93.49	0.05	92.43	93.49	-1.06	86.39	87.85	-1.46
PNC1+^c^ visit	Baseline	51.36	51.96	-0.60	56.00	51.96	4.04	46.67	44.13	2.54
	Endline	79.10	79.00	0.10	76.75	79.00	-2.25*	70.47	70.28	0.19
PNC3+^d^ visits	Baseline	5.03	5.56	-0.53	4.53	5.56	-1.03	2.11	3.91	-1.80
	Endline	16.09	15.15	0.94	12.76	15.15	-2.39	8.81	10.83	-2.02
Modern family planning methods	Baseline	12.13	12.05	0.08	10.74	12.05	-1.31	12.11	15.94	-3.83*
	Endline	29.02	30.35	-1.33	28.65	30.35	-1.70	29.01	35.46	-6.45**

Abbreviations: TTV, tetanus toxoid vaccine; PNC, postnatal care; PBF, performance-based financing; ANC, antenatal care. ^a^At least 4 antenatal care; ^b^At least 2 tetanus doses during pregnancy; ^c^At least one postnatal care visit within 6 weeks; ^d^At least 3 postnatal care visits within 6 weeks. *T* test for differences between PBF2 and PBF1, PBF3 and PBF1 “in 4 regions” and PBF4 and PBF1 “in Boucle du Mouhoun” samples.* *P* < .1, ** *P* < .05, *** *P* < .01.

The random allocation of facilities to the 4 PBF intervention arms resulted in uniform allocation for the majority of the outcome variables intervention arms, and utilization of most maternal care services increased over time with the exception of TTV2+ (Table 8).

####  Regression Analysis

Table 9 summarizes the results of the regression models aimed at estimating the additional benefit of PBF2, PBF3, and PBF4 compared to PBF1 for study component 2. Only PBF4 appeared to produce additional benefits, with significant positive effects on TTV2+ by 13.1pp (*P* < .05) and iron supplementation by 6.2 pp (*P* < .05) over and above PBF1. PBF2 performed worse than PBF1 in terms of its effect on utilization of TTV2+ by 6.8 pp (*P* < .1), while PBF3 had negative additional effects on facility-based delivery by 3.8 pp (*P* < .1) and PNC1+ visit by 6.7 pp (*P* < .1) respectively.

**Table 9:** DID Estimates of the Additional Effects of PBF2-PBF4 Compared With PBF1 on the Utilization of Maternal Health Services

**Outcome variable**	**PBF2 vs. PBF1**		**PBF3 vs. PBF1**		**PBF4 vs. PBF1**	
	**Full Sample**					
	**Beta**	**Robust Standard Error**	**Beta**	**Robust Standard Error**	**Beta**	**Robust Standard Error**
ANC 1sttrimester	-0.036	0.028	-0.007	0.030	-0.064	0.049
ANC4+^a^ visits	-0.026	0.036	-0.018	0.033	-0.031	0.063
TTV2+^b^	-0.068*	0.038	0.054	0.036	0.131**	0.053
HIV testing in pregnancy	0.030	0.039	-0.012	0.036	0.007	0.066
Iron supplementation	-0.010	0.013	0.000	0.012	0.062**	0.023
Facility-based delivery	-0.033	0.021	-0.038*	0.022	0.008	0.049
PNC1+^c^ visit	0.012	0.036	-0.067*	0.035	-0.016	0.053
PNC3+^d^ visits	0.018	0.021	-0.010	0.020	-0.022	0.028
Modern family planning methods	-0.021	0.024	-0.011	0.025	-0.031	0.043

Abbreviations: TTV, tetanus toxoid vaccine; PNC, postnatal care; DID, difference-in-differences; PBF, performance-based financing; ANC, antenatal care. ^a^At least 4 antenatal care; ^b^At least 2 tetanus doses during pregnancy; ^c^At least one postnatal care visit within 6 weeks; ^d^At least 3 postnatal care visits within 6 weeks.* *P* < .1, ** *P* < .05.

Table 10 represents the core of our analysis, as it summarizes the results of the regression models aimed at estimating the additional benefit of PBF2, PBF3, and PBF4 compared to PBF1 by socio-economic subgroup for study component 2. Similar to the overall findings presented in Table 9, the equity measures that accompanied the implementation of PBF did not result in any additional benefit for the poorest 20%, but rather the opposite on certain indicators. PBF2 and PBF3 decreased utilization of facility-based delivery by 11.7 pp (*P* < .05) and 11.8 pp (*P* < .05), respectively among the poorest 20%. In addition, PBF3 decreased utilization of iron supplementation by 7.4 pp (*P* < .05) and modern family planning methods by 12.7 pp (*P* < .05) among the poorest 20%, while PBF4 decreased utilization of PNC1+ visit by 24.1 pp (*P* < .05) among the poorest 20%. The overall positive additional effect of PBF4 on TTV2+ and iron supplementation coverage (seen in Table 9) was present only among the upper 80%, with an increase of 13.7 pp (*P* < .05) and 7.6 pp (*P* < .05) respectively, but not among the poorest 20%.

**Table 10:** DID Estimates of the Additional Effects of PBF2-PBF4 Compared With PBF1 on the Utilization of Maternal Health Services by Wealth Subgroups

**Outcome variable**	**PBF2 vs PBF1**				**PBF3 vs PBF1**				**PBF4 vs PBF1**			
	**Wealth subgroups**											
	**Lowest 20%**		**Upper 80%**		**Lowest20%**		**Upper 80%**		**Lowest 20%**		**Upper 80%**	
	**Beta**	**Robust Standard Error**	**Beta**	**Robust Standard Error**	**Beta**	**Robust Standard Error**	**Beta**	**Robust Standard Error**	**Beta**	**Robust Standard Error**	**Beta**	**Robust Standard Error**
ANC 1sttrimester	-0.071	0.069	-0.031	0.031	-0.037	0.070	-0.006	0.033	0.073	0.122	-0.087	0.059
ANC4+^a^ visits	-0.038	0.082	-0.038	0.038	-0.026	0.082	-0.025	0.037	0.020	0.133	-0.049	0.074
TTV2+^b^	-0.013	0.072	-0.074*	0.040	0.013	0.077	0.074*	0.039	0.099	0.098	0.137**	0.059
HIV testing in pregnancy	0.085	0.088	0.018	0.042	0.041	0.087	-0.023	0.038	-0.064	0.136	0.033	0.067
Iron supplementation	-0.004	0.036	-0.015	0.014	-0.074**	0.036	0.011	0.013	0.036	0.073	0.076**	0.029
Facility-based delivery	-0.117**	0.048	-0.026	0.023	-0.118**	0.056	-0.015	0.023	-0.014	0.083	0.067	0.050
PNC1+^c^ visit	-0.041	0.084	0.028	0.039	-0.103	0.081	-0.050	0.040	-0.241**	0.110	0.014	0.058
PNC3+^d^ visits	0.052	0.052	0.005	0.024	0.076	0.053	-0.024	0.023	-0.069	0.071	-0.016	0.033
Modern family planning methods	-0.022	0.054	-0.019	0.027	-0.127**	0.054	0.021	0.026	0.023	0.103	-0.057	0.049

Abbreviations: TTV, tetanus toxoid vaccine; PNC, postnatal care; DID, difference-in-differences; PBF, performance-based financing; ANC, antenatal care. ^a^At least 4 antenatal care; ^b^At least 2 tetanus doses during pregnancy; ^c^At least one postnatal care visit within 6 weeks; ^d^At least 3 postnatal care visits within 6 weeks.* *P* < .1, ** *P* < .05.

## Discussion

 This study makes a unique contribution to the literature by combining experimental and quasi-experimental elements to investigate not only the overall impact of PBF on maternal health service coverage, but specifically the role of combining equity interventions with standard PBF to reduce existing inequities.

Unfortunately, the results do not correspond to what policy-makers and their development partners had intended to achieve when designing the intervention and even yield opposite results. It is unfortunate that no data on costs of any interventions (PBF, equity measures, gratuité) are available which would have allowed policy-makers to put the results into a better perspective. Nevertheless, appraising findings across our multiple strains of analysis, it appears that while PBF produced modest changes compared to status quo, the implementation of equity interventions did not generate additional benefits compared to PBF alone, neither for women in general nor for the poorest women specifically. In a few selected instances, PBF combined with equity interventions even resulted in worse outcomes than PBF alone. This observation is aligned with some published evidence. For instance, in Cambodia, coupling PBF with maternity vouchers to cover user fees for the poor was also observed not to improve service utilization for the poor.^27^ Two studies from Rwanda, where PBF was coupled with CBHI in the analysis, showed mixed results. One study found that PBF yielded no equity effects,^12^ while the other study detected pro- poor effects for utilization of facility-based deliveries but negative equity effect on use of modern family planning methods among the poor.^8^

Before we attempt to uncover reasons for why PBF did not attain the intended equity effects, we shall briefly comment on our finding regarding facility-based delivery. While an absolute change of 4 percentage points at a significance level of 10% may appear to be negligible, it is in fact remarkable considering the extremely high baseline utilization values, approaching 90%. In addition, we ought to consider that study component 1 was largely underpowered due to the constraints imposed by the small number of clusters, equivalent to 24 districts. Since the least-poor drove changes in utilization of facility-based delivery, it is plausible to assume that PBF might have produced positive changes in quality of service delivery necessary to encourage further utilization among those with means to be receptive to quality improvements. Further research into the impact of PBF on quality of service delivery is needed to verify this hypothesis. The positive change observed on PNC is less striking, since baseline utilization values departed from relatively low levels. Nevertheless, this change is highly relevant given that health systems currently struggle to increase use of PNC services.^28^ The general decline in coverage of TTV2+ from baseline to endline in both PBF and control catchment areas may be due to the fact that by the time the endline data were collected, most women in the catchment areas had received the 5 doses stipulated by government policy and were therefore not eligible to receive additional vaccination. This issue arose as the question in the survey was set to capture new vaccinations rather than overall vaccination coverage.

Understanding the lack of additional benefit produced by the more complex PBF models integrating equity interventions compared to standard PBF requires a closer inspection of the study context. Endline data collection took place between April and June 2017, approximately one year after the launch of the national free healthcare policy targeting women and children.^17^ Given a pregnancy recall period of 24 months, this means that by the time we collected endline data, only a portion of women in our study area had been exempted from payment of user fees for all maternal care services except modern family planning methods, irrespective of whether they lived in the catchment area of a standard PBF facility (PBF1) or in areas with an additional targeting and subsidization of the ultra-poor (PBF2, PBF3) or a CBHI (PBF4) model.

It could be argued that following the launch of the national free healthcare policy, health service use for the specific indicators included in our study among the ultra-poor might have caught up so fast in PBF1 areas due to the removal of the financial barrier to make it impossible for us to detect any effect of the PBF equity interventions which might have been there prior to June 2016. Our analysis, however, clearly indicates that saturation (ie, utilization rates of 100%) was not reached for any of the targeted indicators. In addition, we note that our effect estimation is by no means invalidated by the implementation of the national free healthcare policy for maternal health, since pre- and post-test designs with independent controls and relying on a DID analytical approach are not compromised by presence of group-invariant factors, such as policies launched across all districts in the country simultaneously.^29^ As such, if the national free healthcare policy did bear any effect on service utilization (which we do not know because this is beyond the scope of this study), it is likely to have done so in all PBF models and control districts, not affecting in any way our ability to detect differences between PBF and control districts and across PBF models.

Nevertheless, we need to acknowledge that the introduction of the national free healthcare policy induced policy-makers to adjust the implementation of the equity measures in PBF2 and PBF3. Specifically, qualitative interviews with key stakeholders revealed that following the launch of the national free healthcare policy, the Ministry of Health removed additional compensation to healthcare facilities in PBF2 and PBF3 for all those services which were included in the free healthcare policy benefit package. This means that effectively, by the time we collected endline data, PBF2 and PBF3 were equivalent to PBF1 in terms of incentives related to all maternal care services except modern family planning. This could well have demotivated health providers from seeking innovative strategies for reaching out to provide the poor with the needed services.^30^ It ought to be noted, however, that the introduction of the national free healthcare policy only touched the assumed financial mechanisms of the equity components, while effects of the sensitization mechanisms activated by the targeting exercise should have remained constant in PBF2 and PBF3 facility catchment areas only, but not in PBF1 facility catchment areas. As such, the introduction of the free national healthcare policy could have diluted, but not fully removed, the effect of the equity measures, had there been one in the first place.

The fact that we detected a negative effect of PBF2, PBF3, and PBF4 compared to PBF1 on selected indicators, especially when considering the stratified analysis looking only at the poorest, is worrisome. However, this appears to corroborate existing evidence pointing at the presence of unintended consequences related to the implementation of the community-based targeting and related subsidized program ^30^ and at general challenges related to the implementation of the overall PBF program in the country.^31,32^ Appraising our current findings in light of existing literature suggests that combining PBF and equity interventions into a single intervention might have resulted in a level of complexity not easily manageable for front-line healthcare providers, ultimately leading to effects contrary to the ones that had been anticipated. For example, evidence shows that health providers introduced ceilings to services offered to the ultra-poor as a way of adapting to the complexity of the PBF interventions and to the long delays in receiving incentive payments, which created financial difficulties for health workers.^30^

Albeit worrisome, the results of our analysis are not per se surprising as they align with PBF evidence from other settings as well as with prior research assessing the impact of earlier targeted exemption policies in Burkina Faso. For instance, the national obstetric care policy implemented from 2007 to 2016 failed to reach the poorest women effectively.^33^ A recently published study indicated that lack of fidelity in implementing exemption policies may be due to providers’ lack of adequate knowledge in the first place.^34^ This suggests a need to educate providers on the purpose and procedures of a given policy to transform them into real agents of change, since poor communities are not sufficiently empowered to overcome all relevant barriers to access in response to a single targeting mechanism. Further qualitative inquiry is needed to unravel if and to what extent providers’ understanding of the targeted exemptions represented a barrier to the effective implementation of equity interventions in Burkina Faso.

In addition, it is possible that mere removal of user fees through targeting was insufficient to enable very poor people to seek care. Prior evidence from the region and the country specifically clearly points at the presence of important non-financial barriers to access.^33,35^ For example, inequities existed in facility-based delivery due to distance to catchment primary health facility, literacy, parity and religion.^21^ There were also inequities in utilization rates for ANC4+ visits due to distance to catchment primary health facility, literacy, parity, religion and marital status and for PNC1+ visit due to distance to catchment primary health facility, age and religion.^21^ Since the PBF program did not address these other sources of inequity, other than household wealth, arguably they still constituted barriers to uptake of essential maternal health services by the poorest women. Still, further research is needed to unravel why in some settings equity interventions are not effective in narrowing equity gaps while in others, such as Tanzania,^10^ combining PBF and targeted exemptions resulted in greater service use among the poor in public health facilities. This is in line with Renmans et al, who have observed that although PBF has received increasing attention, a lot remains unknown about the exact mechanisms triggered by PBF arrangements.^36^ As such, they have called for more research to examine the exact mechanisms through which not only incentives, but also ancillary components operate. Such knowledge is necessary to understand and appreciate the effectiveness, desirability and appropriateness of PBF as a possible tool towards health systems strengthening in LMICs.

###  Methodological Considerations

Our study is not without limitations. First, since the intervention took place within a real-life setting, we cannot rule-out that other interventions with similar objectives took place alongside PBF, especially in control districts. Hence, we cannot estimate the extent to which our comparator really reflects status quo utilization rates. Second, we need to acknowledge the fact that women identified in our study as the poorest do not exactly match the ones identified by the community-based targeting procedure of the program as such. Hence, the reader ought to be aware that our findings illustrate the impact on the lowest quintile in general and not on targeted individuals specifically. Parallel research efforts based on a different dataset are ongoing to look at the impact of the equity interventions specifically on targeted individuals. Third, the power to detect impact in study component 1 was limited by the relatively low number of clusters. This limitation was noted well in advance, when the overall PBF impact evaluation study was being designed, but financial and policy challenges made it impossible to increase the number of clusters, and hence it was agreed amongst all key stakeholders to live with this limitation. Fourth, the purposive selection of the districts represents a potential threat to external validity, more specifically to the generalizability of the results emerging from study component 1. Precisely, the purposive selection of the districts does not allow us to make inferences about the possible effects of PBF in districts with higher baseline values, hence we need to exercise caution in generalizing the results of this study to other contexts. This purposive selection, however, does not represent a threat in terms of the internal validity of the DID analysis, since it does not violate the basic assumptions of the DID model.^29,37^ Fifth, as discussed extensively earlier, the modifications which were operated to the PBF design following the introduction of the national free healthcare policy could have potentially contributed to diluting, but not eliminating, the effect of PBF, had there been one in the first place.

## Conclusion

 PBF is being implemented in LMICs and sub-Saharan Africa in particular as a response to weak health systems performance. Although rapidly growing, evidence regarding its effectiveness is still very mixed. Evidence regarding PBF equity impacts on use of health services and maternal health is particularly scarce and, when available, it is mixed, in some cases conflicting. Our results indicate that even well-designed PBF interventions which integrate explicit equity components are not sufficient to overcome inequities in health service use. As such, our results confirm the need for additional interventions reaching beyond the financial realm to ensure access to care by the ultra-poor. In addition, our findings suggest that changing policy environments inevitably affect the way an intervention, in this case PBF combined with equity measures, is carried out and hence should be explicitly acknowledged when appraising effects.

 Lastly, we would like to reiterate the importance of carefully monitoring and measuring the equity impact of interventions targeted at improving access and quality of service delivery as an integral element of SDG 3. The experience of the PBF program in Burkina Faso provides a clear illustration of how even well-intended and accurately designed interventions may fail to achieve their objective due to a variety of contextual elements that shape implementation in unattended manners. As such, greater attention to context and competing policies ought to be paid in designing strategies that aim at building synergies between supply and demand to overcome existing inequities.

## Acknowledgements

 This work was supported by the World Bank through the Health Results Innovation Trust Fund (HRITF). We thank the Programme d’Appui au Développement Sanitaire (PADS) for financial support for data collection given to Centre Muraz. We also thank Aurélia Souares, Nobila Ouedraogo, Hien Herve, and Herman Badolo for successfully coordinating baseline and endline data collection, respectively and all Muraz staff and interviewers for the fieldwork.

## Ethical issues

 The ethical clearance for the study was obtained from the Ethical Committee of Heidelberg University (S-272/2013) and from the National Ethics Committee in Burkina Faso (N° 2013-7-066 and N° 2015-5-071).

## Competing interests

 This work was supported by the World Bank through HRITF, which was administered by the Medical Faculty of the University of Heidelberg. The World Bank was engaged in the overall design of the intervention and the impact evaluation, but had no role in data collection, data management, data analysis and interpretation, preparation, review and approval of the manuscript. MDA was the Principal Investigator of the IE, but received no direct compensation from the World Bank. TM is a doctoral student at the University of Heidelberg, who was not part of the IE and contributed his own time towards conception of the study, data analysis and interpretation, and drafting of the manuscript. JL, MS, and SB were fully or partially funded by the HRITF grant to the University and worked on the IE (data collection, management, and reporting to the World Bank), but received no direct payment by the Bank nor any compensation for manuscript preparation (which occurred outside the framework of the contract with the World Bank). SS and HH are employees of Centre MURAZ, but were not directly funded by the HRITF grant allocated to Centre MURAZ for data collection. SH is a World Bank employee based in Yaoundé, Cameroon and contributed to the data collection and management in the context of the IE, but participated in writing of this paper independently of his professional engagement. The views reported in this paper represent the views of the authors exclusively and not those of the funding agency.

## Authors’ contributions

 TM, MDA, and JL conceived the study. JL, MDA, SB, SMAS, SH, and HH contributed to survey design and data collection. TM undertook data analysis with support from JL, MDA, and MH. All authors contributed to the interpretation of the results. TM drafted the manuscript with contributions from all authors.

## Authors’ affiliations

^1^Heidelberg Institute of Global Health, Medical Faculty and University Hospital, Heidelberg University, Heidelberg, Germany. ^2^Department of Global Health and Development, London School of Hygiene & Tropical Medicine, London, UK. ^3^The World Bank, Yaoundé, Cameroon. ^4^Centre MURAZ, Bobo-Dioulasso, Burkina Faso. ^5^UFR/ST, Université Nazi Boni, Bobo-Dioulasso, Burkina Faso.
